# AI-assisted reliability-based design framework for tunnel concrete linings in weak rocks

**DOI:** 10.1038/s41598-026-44903-9

**Published:** 2026-03-20

**Authors:** Jafar Khani, Hamid Reza Nejati, Kamran Goshtasbi, Sina Rostamabadi, Arsalan Mahmoodzadeh

**Affiliations:** 1 Department of Rock Mechanics, School of Engineering, Tarbiat Modares University, Tehran, Iran; 2 Center of Research and Strategic Studies, Lebanese French University, Erbil, Iraq

**Keywords:** Reliability-based design, Convergence–confinement method, Artificial intelligence, Tunnel lining, Weak rock, Engineering, Materials science, Mathematics and computing

## Abstract

Reliability-based design (RBD) of tunnel concrete linings in weak rocks is challenging due to the uncertainty of geomechanical parameters and the ground–support interaction. This study introduces a transparent and efficient framework that integrates three key components: (1) implicit ground–support equilibrium analysis based on the Convergence–Confinement Method (CCM), (2) probabilistic evaluation of the safety factor ($\:SF$), reliability index ($\:\beta\:$), and failure probability ($\:{P}_{f}$) and (3) a data-driven surrogate model based on artificial intelligence for rapid parametric analysis. The uncertainties of the rock mass and concrete linings are treated through Monte Carlo sampling, and the outcomes are benchmarked against the First-Order Reliability Method (FORM) to verify accuracy and potential bias. The results are transformed into decision-making charts linking the thickness and compressive strength of concrete linings to target $\:\beta\:/{P}_{f}$ levels, thus providing risk-consistent objectives instead of a fixed safety factor criterion. Two types of linings are considered: conventional concrete (with an average uniaxial compressive strength of 20 MPa) and fiber-reinforced reactive powder concrete (FRPC, with an average uniaxial compressive strength of 65 MPa, developed by the research team). The findings demonstrate that increasing thickness and material quality significantly reduce $\:{P}_{f}$, achieving the reliability thresholds required for final tunnel support (2E-5). This effect is more critical at smaller thicknesses and with lower-quality materials. The use of FRPC also leads to a considerable reduction in $\:{P}_{f}$ at intermediate thicknesses, making it an efficient option when construction constraints limit thickness increase. The surrogate model successfully reproduces probabilistic trends with high consistency and slight conservatism. Ultimately, the integrated CCM–reliability–artificial intelligence framework bridges the gap between deterministic design and risk management, delivering economical and resilient tunnel lining designs in weak rocks.

**Supplementary Information:**

The online version contains supplementary material available at 10.1038/s41598-026-44903-9.

## Introduction

The design of underground structures, particularly tunnels, has always been accompanied by multiple challenges. One of the most critical challenges is the uncertainty in estimating ground properties and loading conditions, which can significantly affect the performance and stability of the structure. In this context, two main approaches are commonly adopted in design: deterministic methods and probabilistic methods. In deterministic approaches, design parameters are assumed to be fixed values without accounting for uncertainties. This simplification may lead to unrealistic or overly conservative designs, since natural variability in geotechnical parameters is neglected. Conversely, probabilistic methods incorporate statistical distributions of parameters and risk analysis, resulting in designs that are more realistic and better suited to uncertain conditions. The fundamental distinction between deterministic and probabilistic approaches lies in how they address uncertainty. Deterministic methods, rooted in early studies such as those of Terzaghi^1^, assume that input parameters such as rock strength and applied loads are constant and known values. While this assumption simplifies calculations, it fails to capture the inherent variability of data and its effects on structural stability. In contrast, probabilistic approaches, which have evolved since the 1970 s through the statistical models developed by researchers such as Hoek and Brown^2^, explicitly incorporate uncertainties into computations through probability distributions. This approach enables the estimation of failure probability (P_f_) and the determination of the safety margin, thereby providing more robust and economical designs. Classical studies such as those by Bieniawski^3^ emphasized deterministic methods for tunnel stability analysis; however, with technological advancements and the demand for higher accuracy, research by Einstein and Schwartz^4^ highlighted the importance of probabilistic methods in underground risk assessment. In subsequent decades, the development of numerical and statistical models, as seen in the work of Hudson and Feng^5^, demonstrated that integrating Ground Reaction Curves (GRC) with reliability-based design could provide deeper insights into rock–concrete interaction. These studies revealed that deterministic approaches, by ignoring parameter variability, may lead to overly optimistic or conservative outcomes, whereas probabilistic methods, by exploring a wide range of scenarios, enable more informed decision-making. Estimating the type and extent of support required for excavation stabilization, as well as predicting the final wall convergence near the tunnel face, has consistently been one of the central concerns in tunnel design. Several methods have been employed for such analyses, among which the Convergence–Confinement Method (CCM) has been widely adopted. Most analyses based on CCM have been conducted deterministically^6–9^. Nevertheless, due to the inherent uncertainties in rock mass properties, probabilistic analyses are considered a more rational approach, as they explicitly demonstrate the effects of uncertainty and can differentiate between critical and less significant sources of variability.

Analysis of circular tunnels under hydrostatic stress, despite its relative simplicity, continues to serve as an effective tool for evaluating the reliability of underground structural performance. Li and Lu^10^, employing the First-Order Reliability Method (FORM), calculated the reliability index of a circular tunnel subjected to hydrostatic stress and examined the influence of normal and lognormal distributions for random variables. Their results indicated that incorporating negative correlations between strength parameters such as cohesion and friction angle increases the reliability index, while neglecting such correlations may lead to unnecessarily conservative assessments. Furthermore, a comparison between FORM and Monte Carlo simulations demonstrated that the proposed method provides an adequate approximation of the probability density function of the outputs and can reliably replace computationally expensive numerical simulations. In recent years, the application of reliability analysis methods for assessing the performance of tunnel support systems in circular excavations has attracted growing attention. A key issue in this context is the rigorous evaluation of the interaction between the rock mass and the support system, which is influenced by multiple random parameters, including rock mechanics properties, environmental loadings, and the geometric and strength characteristics of the lining. Under such conditions, numerical approaches such as the Response Surface Method (RSM) have been employed as effective tools to simplify probabilistic analysis. Lu, Sun, and Lu^11^ developed an efficient framework for reliability analysis of ground–support interaction by combining RSM with FORM and SORM. In their study, an elastoplastic model was adopted for the rock mass and a linear elastic model for the support lining. The limit state function was defined based on three performance criteria: lining capacity, allowable tunnel convergence, and sufficient bolt anchorage length, which were then used to evaluate the failure probability of the support system. The results revealed that RSM substantially reduces computational time while maintaining high accuracy in approximating the limit state function, with satisfactory agreement compared to Monte Carlo simulation outcomes. It was also emphasized that the installation location of the support system has a significant impact on the probability of failure and should therefore be optimally determined.

Complex constitutive models, such as the Elastic-Strain-Softening (ESS) model, have gained increasing importance in tunnel stability analysis owing to their ability to capture the gradual mechanical degradation of medium-quality rock masses. Song et al. by employing methods such as FORM and SORM, performed a probabilistic analysis of a circular tunnel in a rock mass exhibiting Elastic-Strain-Softening (ESS) behavior and demonstrated that parameters such as support pressure and in-situ stresses have a significant influence on the probability of failure. This study represented an important step toward reliability-based design of tunnel supports in non-elastic rock masses^12^. In tunnel support design, simultaneously accounting for safety and cost-effectiveness under multiple failure modes has always been a critical challenge. In this regard, Lü et al. proposed a reliability-based design optimization (RBDO) framework using the Response Surface Method (RSM) to evaluate tunnel supports in rock masses under various failure modes. Their results indicated that design variables such as concrete thickness and installation location have a profound effect on the reliability index and overall cost, and that by adjusting these parameters, an economical yet safe design can be achieved^13^.

When tunnel design in rock masses is accompanied by high levels of geomechanical uncertainty, the observational method is recognized as a dynamic tool for updating designs during construction. Bjureland et al. by integrating reliability-based design with the observational method, proposed a comprehensive framework for evaluating and improving tunnel support safety. Through deformation analysis and Bayesian updating, they enabled the prediction of future tunnel behavior and demonstrated that this approach can effectively reduce risk and enhance decision-making throughout different excavation stages^14^. In the design of tunnel supports using concrete linings, accurate statistical characterization of their mechanical properties plays a critical role in reliability-based analyses. Bjureland et al. by analyzing data from the Stockholm urban tunnel project, derived statistical distributions for parameters such as thickness, cohesion, flexural strength, and compressive strength of concrete linings. By introducing a conditional correlated model, they showed that employing these distributions in numerical analyses allows for more accurate estimation of failure probability and contributes to safer and more economical designs^15^. Reliability-based design optimization (RBDO) in rock engineering has consistently faced computational challenges due to the nonlinear and multidimensional behavior of underground structures. Zhao proposed a practical and efficient approach by integrating High Dimensional Model Representation (HDMR) with Multiplicative Dimensional Reduction Method (MDRM). This method, combined with the First-Order Second-Moment (FOSM) algorithm, enables the calculation of the reliability index and optimization of support parameters with high accuracy and reduced computational effort, without the need for repeated simulations. The incorporation of Excel Solver within this framework highlights its potential for industrial application in real tunneling projects^16^. For the reliability analysis of deep rock tunnels, Silva et al. introduced a simplified approach based on the First-Order Reliability Method (FORM) and Monte Carlo Simulation (MCS). In their study, the elastoplastic behavior of the rock mass was modeled using the Mohr–Coulomb criterion in conjunction with the Convergence–Confinement Method. The results showed that increasing support pressure and lining thickness significantly reduces the probability of failure. A comparison between two tunnels with different geotechnical conditions further underscored the importance of correlations among random variables and the appropriate selection of the performance function in reliability analyses^17^. Empirical rock mass classification systems, such as the Q-system, are effective tools for preliminary tunnel stability assessments; however, their application in reliability analysis has been relatively limited. Lu et al. by combining the Q-based empirical approach with FORM, proposed a methodology for evaluating instability probability in rock tunnels. In this framework, critical strain was treated as a random variable, and the limit state function was explicitly influenced by geotechnical uncertainties. A case study of the Shimizu Tunnel in Japan, validated against Monte Carlo simulations, demonstrated that the Q-based empirical model provides both realistic and computationally efficient estimates of the reliability index, serving as a complementary tool to numerical methods in design^18^. In rock tunneling projects with concrete linings, defining reliability-based thresholds represents a key component of the observational design framework. Spross et al. by using subset simulation and finite element modeling, proposed a method for establishing deformation thresholds of concrete linings, ensuring that the probability of failure remains controlled when the thresholds are not exceeded. A case study from the Stockholm Bypass project revealed that adopting such thresholds not only optimizes initial design but also allows for reduced conservatism and improved geotechnical risk management when coupled with field monitoring^19^. Reliability-based tunnel support design using Ground Reaction Curves (GRC) requires accurate modeling of the post-failure behavior of rock masses. Zhao et al. introduced a novel numerical algorithm based on the self-similarity method to derive ground reaction curves in softening rock masses, developed upon the generalized Hoek–Brown failure criterion. By discretizing the plastic zone into concentric rings and applying the fourth-order Runge–Kutta method, the algorithm is capable of predicting stress distribution, displacement, and the extent of the softened zone with high accuracy. Parametric investigations indicated that the parameter a in the Hoek–Brown criterion exerts a pronounced influence on failure extent and excavation boundary displacement, particularly under low support pressures^20^.

Previous studies have revealed that incorporating all mechanical and strength parameters of the ground and support system (concrete lining) in a probabilistic manner within the Convergence–Confinement design framework is subject to certain limitations. To address this challenge, the present study develops an innovative spreadsheet-based tool in Microsoft Excel to perform Convergence–Confinement analytical calculations. In addition to allowing all design parameters to be introduced as probabilistic variables, this new code also enables precise computation of the design safety factor using the Solver module in Excel. An Excel-based macro-enabled spreadsheet was developed to implement the Convergence–Confinement Method (CCM) using an implicit solution strategy, providing a rapid and user-friendly computational environment. This tool enables efficient evaluation of the safety factor under both deterministic and probabilistic conditions, facilitating transparent and reproducible application of the CCM equations.

In conventional reliability analyses for tunnel design, assumptions are often made that lead to the use of predefined and imprecise limit state functions. Therefore, in this study, by employing artificial intelligence together with the accurate results obtained from the newly developed code, a novel limit state function for the concrete support system in tunnel stability design was derived. To achieve this objective, the output generated by the macro-enabled Excel spreadsheet was used to construct the training, testing, and validation datasets for the GEP-based surrogate model. Through this process, an explicit predictive equation was derived, enabling accurate estimation of the safety factor.

Another key contribution of this research is the establishment of a meaningful relationship between the safety factor and the probability of failure in tunnel reliability analyses, such that an exact equivalent safety factor can be calculated and reported for any given failure probability. This implies that the proposed approach enables rapid and accurate prediction of the tunnel stability safety factor, while also allowing the corresponding failure probability to be estimated directly, without the need for additional reliability analyses.

All computations were carried out for two categories of concrete support systems. The first category corresponds to conventional concrete linings with an average uniaxial compressive strength of 20 MPa, while the second involves fiber-reinforced reactive powder concrete (FRPC) with an average uniaxial compressive strength of 65 MPa, which was designed and developed by the authors of this study. This advanced type of concrete was specifically engineered by applying the principles of reactive powder concrete to the production of high-strength sprayed concrete. The results demonstrate that FRPC linings enable their use as final tunnel support systems with extremely low probabilities of failure.

## Methodology

The research methodology was designed with the objective of establishing a comprehensive framework for the reliability-based design of tunnel support systems with concrete linings in weak rocks. The overall procedure consists of four main steps: (1) analytical formulation based on the Convergence–Confinement Method (CCM), (2) probabilistic reliability analysis using the First-Order Reliability Method (FORM) and Monte Carlo simulation, (3) development of a computational engine in Microsoft Excel incorporating the Solver functionality and macro programming to explicitly solve implicit equations, and (4) derivation of a surrogate limit state function using artificial intelligence to enable rapid parametric analyses.

The interaction between the rock mass and the tunnel support system was modeled using the Convergence–Confinement Method (CCM). The mechanical behavior of the rock was characterized based on the generalized Hoek–Brown failure criterion, while the concrete lining was modeled as elastic up to its compressive capacity. The Ground Reaction Curve (GRC) and the Support Capacity Curve (SCC) were explicitly formulated, and the equilibrium condition was obtained through iterative solutions using the Solver functionality within the Excel-based computational spreadsheet. To overcome the common limitations associated with scaled parameters, the fundamental equations were reformulated so that the system could be solved solely in terms of the original mechanical and geometric variables.

All parameters of the rock mass and the concrete lining—including the uniaxial compressive strength of rock and concrete, Hoek–Brown constants $\:m$, $\:s$, and $\:a$, Young’s modulus, Poisson’s ratio, tunnel radius, and lining thickness, were treated as random variables with appropriate probabilistic distributions. The reliability analysis was then carried out using two complementary approaches:

- Implementation of Monte Carlo simulation through macro programming to propagate uncertainties and directly compute the probability of failure ($\:{P}_{f}$) based on thousands of samples.
- Application of the FORM algorithm to calculate the reliability index ($\:\beta\:$) at the design point.

A new Excel-based code was developed that, leveraging Solver and macro programming, automatically solves the implicit CCM relations. For each random input realization, the equilibrium point and the pressure-based safety factor ($\:{SF}_{p}$) were computed, and the results were stored together with the corresponding input parameters. The safety factor computed in this study is based on the pressure- controlled safety factor (SF_p_), and other types of safety factors, such as displacement or strain-based factors, were not considered. The probability of failure was then obtained as the proportion of cases for which $\:{SF}_{p}<1$. This large dataset provided the basis for statistical analyses and for training the artificial intelligence model.

To accelerate parametric analyses, a surrogate model based on machine learning was trained to provide an explicit nonlinear relationship between the input variables and the pressure-based safety factor ($\:{SF}_{p}$). Compared to the probabilistic mean values, this model exhibited slightly conservative behavior, while successfully reproducing the results with a very high correlation coefficient ($\:R^2$ greater than 0.99).

To investigate the effect of material quality, two types of concrete linings were considered in the analyses: conventional concrete with an average uniaxial compressive strength of approximately 20 MPa, and fiber-reinforced reactive powder concrete (FRPC) with an average uniaxial compressive strength of about 65 MPa. Laboratory results for FRPC indicated that this material possesses both high compressive strength and notable ductility, making it suitable for use as a final lining with very low risk.

Finally, the results obtained from the spreadsheet and the artificial intelligence model were compared with the FORM analysis. This comparison revealed that simpler deterministic-based solutions may be overly optimistic under critical conditions, whereas the probabilistic FORM analysis provides greater capability in realistically capturing risk fluctuations. The subsequent section describes the stages and components of the research in detail.

## Materials and methods

### Convergence–Confinement Method (CCM)

One of the most effective analytical approaches for evaluating the interaction between the rock mass and the support system around tunnels is the Convergence–Confinement Method (CCM). This method, by accounting for the gradual changes in stress and strain distributions caused by excavation face advance, enables a more accurate assessment of the loads transferred to the support system. Within this framework, three fundamental components are defined: the Longitudinal Displacement Profile (LDP), the Ground Reaction Curve (GRC), and the Support Characteristic Curve (SCC). The interaction of these three components governs the final load imposed on the support.

**Fig. 1:**
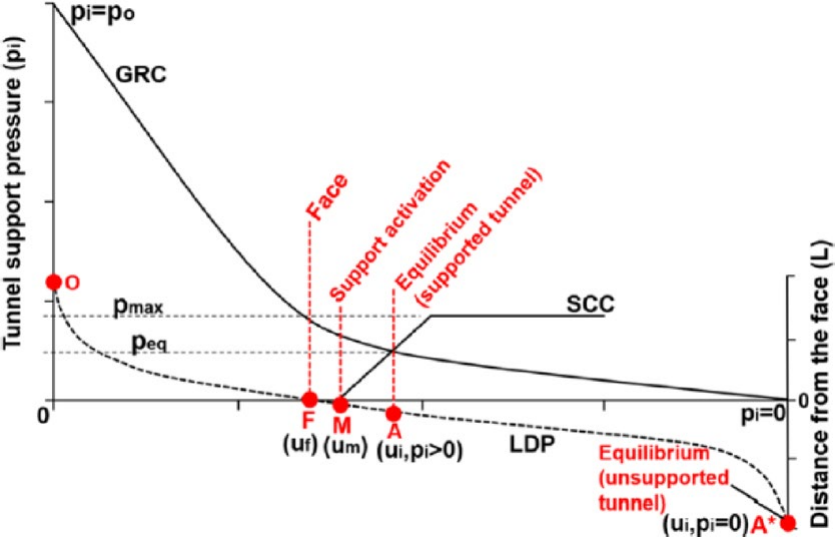
General view of the ground–support interaction diagram^21^.

Modeling the mechanical behavior of the rock mass based on the Hoek–Brown failure criterion, particularly in jointed rocks, is an integral part of the rigorous application of this method. This criterion, by adjusting the strength properties of intact rock samples, facilitates the estimation of reduced rock mass strength at field scale^7^. The schematic representation of the LDP, GRC, and SCC is shown in Fig. 1^21^.

For probabilistic analysis of the aforementioned diagram, it is necessary to separate it into two distinct components and examine each one independently.

#### Ground Reaction Curve (GRC)

This curve is a function of the mechanical properties of the rock mass, including the elastic modulus ($\:E$), Poisson’s ratio ($\:\nu\:$), uniaxial compressive strength ($\:{\sigma\:}_{cm}$), and other geomechanical parameters. Since all of these parameters are inherently random and follow probabilistic distributions, the GRC itself is intrinsically a probabilistic curve and therefore not unique. As a result, instead of a single deterministic curve, a set of GRCs can be generated in accordance with the statistical variability of the input parameters, reflecting the uncertainty in the behavior of the rock mass surrounding the excavation. Two common approaches can be employed for modeling and plotting this curve:

- The first approach is based on the studies of Duncan–Fuma. In this method, the GRC is expressed as a function of the parameters: elastic modulus ($\:E$), internal friction angle ($\:\phi\:$), Poisson’s ratio ($\:\nu\:$), and uniaxial compressive strength ($\:{\sigma\:}_{cm}$). These parameters are calculated using the Geological Strength Index (GSI) of the rock mass.
- The second approach is based on the studies of Carranza-Torres. In this method the E, ν, σ_cm_, GSI, dilatancy angle ($\:\psi\:$), Hoek-Brown constants ($\:{m}_{i}$), Disturbance Factor ($\:D$), dimensionless parameters of the rock mass $\:{m}_{b}$, $\:s$, and $\:a$ are employed.

In this study, the second approach, namely that of Carranza-Torres, is adopted. In this approach, the GRC is calculated and plotted according to the following steps and equations:

First, the two parameters of scaled internal pressure ($\:{P}_{i}$) and scaled in-situ stress ($\:{S}_{o}$) are calculated using Eqs. (1) and (2):

1 $$\:{P}_{i}=\frac{{p}_{i}}{{m}_{b}\:\:{\sigma\:}_{ci}}+\frac{s}{{m}_{b}^{2}}$$

2 $$\:{S}_{o}=\frac{{\sigma\:}_{o}}{{m}_{b}\:\:{\sigma\:}_{ci}}+\frac{s}{{m}_{b}^{2}}\:\:\:\:\:\:\:\:$$

Then, the scaled critical internal pressure ($\:{P}_{i}^{cr}$) is calculated using Eq. (3):

3 $$\:{P}_{i}^{cr}=\frac{1}{16}{\left[1-\sqrt{1+16\:{S}_{o}}\right]}^{2}$$

The actual (unscaled) internal pressure is obtained from Eq. (4):

4 $$\:{p}_{i}^{cr}=\left[{P}_{i}^{cr}-\frac{s}{{m}_{b}^{2}}\right]\:{m}_{b}\:\:{\sigma\:}_{ci}$$

If $\:{p}_{i}\ge\:{p}_{i}^{cr}$, the rock deformation will be elastic, which is obtained from Eq. (5):

5 $$\:{u}_{r}^{el}=\frac{{\sigma\:}_{o}-{p}_{i}}{2{G}_{rm}}R$$

And $\:{p}_{i}<{p}_{i}^{cr}$, the deformation will be plastic. To calculate this deformation, the radius of the plastic zone is first determined using Eq. (6), and then the plastic deformation of the rock (assuming a zero-dilation angle) is computed using Eq. (7):

6 $$\:{R}_{pl}=R\:\mathrm{e}\mathrm{x}\mathrm{p}\left[2\left(\sqrt{{P}_{i}^{cr}-{P}_{i}}\right)\right]$$

7 $$\:{u}_{r}^{pl}=R\frac{{\sigma\:}_{o}-{p}_{i}^{cr}}{2{G}_{rm}}\left[\frac{1-2\upsilon\:}{2}\frac{\sqrt{{P}_{i}^{cr}}}{{S}_{o}-{P}_{i}^{cr}}+1\right]{\left(\frac{{R}_{pl}}{R}\right)}^{2}+\frac{1-2\upsilon\:}{4\left({S}_{o}-{P}_{i}^{cr}\right)}{\left[\mathrm{ln}\left(\frac{{R}_{pl}}{R}\right)\right]}^{2}-\frac{1-2\upsilon\:}{2}\:\frac{\sqrt{{P}_{i}^{cr}}}{{S}_{o}-{P}_{i}^{cr}}\left[2\mathrm{ln}\left(\frac{{R}_{pl}}{R}\right)+1\right]$$

In all the above equations, the variables defining the ground properties—such as $\:E$, $\:\nu\:$, $\:\psi\:$, $\:{\sigma\:}_{cm}$, $\:GSI$, $\:{m}_{i}$, $\:{m}_{b}$, $\:s$, and $\:a$— are all treated as probabilistic variables; therefore, all the aforementioned relations can be solved in a probabilistic manner.

#### Support Characteristic Curve (SCC)

This curve is a function of the parameters: elastic modulus ($\:{E}_{c}$), lining thickness ($\:{t}_{c}$), and uniaxial compressive strength of the support system ($\:{{\upsigma\:}}_{cc}$). These properties are also non-deterministic and are all represented by probabilistic distributions. Consequently, the SCC is inherently a probabilistic curve and not unique. The Support Characteristic Curve (SCC) for concrete linings is expressed using two parameters: the maximum supportable pressure ($\:{p}_{s}^{max}$) and the stiffness of the lining ($\:{\mathrm{k}}_{s}$), which are calculated using Eqs. (8) and (9):

8 $$\:{p}_{s}^{max}=\frac{{{\upsigma\:}}_{cc}}{2}\:\left[1-\frac{{\left(R-{t}_{c}\right)}^{2}}{{R}^{2}}\right]$$

9 $$\:{\mathrm{k}}_{s}=\frac{{E}_{c}}{\left(1+{\upsilon\:}_{c}\right)R}\:\frac{{{R}^{2}-\left(R-{t}_{c}\right)}^{2}}{{\left(1-2{\upsilon\:}_{c}\right)R}^{2}+{\left(R-{t}_{c}\right)}^{2}}$$

The values of $\:{E}_{c}$, $\:{{\upsigma\:}}_{cc}$, $\:{t}_{c}$, and ν follow probabilistic distributions; therefore, both $\:{p}_{s}^{max}$ and $\:{\mathrm{k}}_{s}$ are also probabilistic in nature.

Finally, by plotting the ground reaction curve and the support characteristic curve (assuming $\:{u}_{r}^{o}$​ is known), the ground pressure acting on the support system at the equilibrium point ($\:{p}_{s}^{D}$​) can be calculated using Eq. (10). At the moment of equilibrium between the ground and the support system, their deformations are equal:

10 $$\:{p}_{s}^{D}=\:\:{\mathrm{k}}_{s}\:({u}_{r}^{D}-{u}_{r}^{o})$$

Finally, to define the safety factor in the convergence–confinement method, Eq. (11) is used:

11 $$\:{SF}_{p}=\frac{{p}_{s}^{max}}{{p}_{s}^{D}}$$

#### Failure Probability Function in the Convergence–Confinement Method (CCM)

In classical definitions, the failure probability function is defined such that the safety factor falls below unity. Accordingly, the failure probability function ($\:G$) can be expressed as Eq. (12):

12 $$\:G=SF-1=\frac{{p}_{s}^{max}}{{p}_{s}^{D}}-1$$

To calculate the limit state function, Eq. (11) must first be evaluated. In this relation, the value of $\:{p}_{s}^{max}$ can be obtained from Eq. (8). However, for determining $\:{p}_{s}^{D}$ (an internal pressure lower than the critical internal pressure), Eq. (10) must be used. Assuming that $\:{\mathrm{k}}_{s}$ (from Eq. (9)) and $\:{u}_{r}^{o}$ (by assigning an initial assumption, e.g., equal to the critical elastic deformation or 30% greater) are known, the value of $\:{u}_{r}^{D}$ should be derived from the plastic deformation equation of the rock mass (Eq. (7)). This, in turn, requires calculation of the plastic zone radius around the tunnel ($\:{R}_{pl}$) from Eq. (6). As can be seen, in order to compute Eq. (6), the value of $\:{P}_{i}$ must be determined using Eq. (1) with the given $\:{p}_{i}$. In this case, $\:{p}_{i}$ is exactly the same as $\:{p}_{s}^{D}$. Therefore, solving the problem in this way requires an implicit and iterative solution, which cannot be obtained explicitly. A new approach for solving this problem is presented in Sects. 3–3.

### First-Order Reliability Method (FORM)

Reliability analysis examines the correlation between the loads imposed on a system and its inherent capacity to withstand them^22^. Hasofer and Lind introduced a technique termed geometric reliability, commonly referred to as the First-Order Reliability Method (FORM), to evaluate system reliability^23^. The matrix representation of the FORM approach for normally distributed and uncorrelated parameters has been detailed in several studies, notably by Hasofer and Lind^23^ and Ditlevsen^24^. Low and Tang developed a practical framework that enables the computation of the reliability index through the use of spreadsheet platforms and optimisation techniques^25^. Within this framework, the reliability index is reinterpreted through an elliptical expansion in the original space of fundamental random variables. The reliability index is formulated as shown in Eq. (13)^25^:

13 $$\:\beta\:=\underset{{x\epsilon F}}{\mathrm{min}}\sqrt{{\left(\frac{{x}_{i}-{\mu\:}_{i}}{{\sigma\:}_{i}}\right)}^{T}{R}^{-1}\left(\frac{{x}_{i}-{\mu\:}_{i}}{{\sigma\:}_{i}}\right)}$$

In this formulation, $\:{x}_{i}$ represents the original normal variable, $\:R$ is the correlation matrix of the input parameters, $\:{\mu\:}_{i}$ and $\:{\sigma\:}_{i}$ denote the mean and standard deviation of the random variable, respectively, $\:i$ is the index of the random variables, and $\:F$ represents the failure domain. In cases where the input parameters are correlated and follow non-normal distributions, their equivalent normal means and standard deviations must be used, and the reliability index $\:\beta\:$ can be calculated according to Eq. (14)^26^:

14 $$\:\beta\:=\underset{{x\epsilon F}}{\mathrm{min}}\sqrt{{\left(\frac{{x}_{i}-{{\mu\:}_{i}}^{N}}{{{\sigma\:}_{i}}^{N}}\right)}^{T}{R}^{-1}\left(\frac{{x}_{i}-{{\mu\:}_{i}}^{N}}{{{\sigma\:}_{i}}^{N}}\right)}$$

In this expression, $\:{{\mu\:}_{i}}^{N}$ and $\:{{\sigma\:}_{i}}^{N}$ denote the equivalent normal mean and equivalent normal standard deviation, respectively, for a random variable with a non-normal distribution. The equivalent normal mean and standard deviation for non-normal random variables can be computed using the two-parameter equivalent normal transformation proposed by Rackwitz and Flessler^27^, or other similar transformation techniques.

**Fig. 2:**
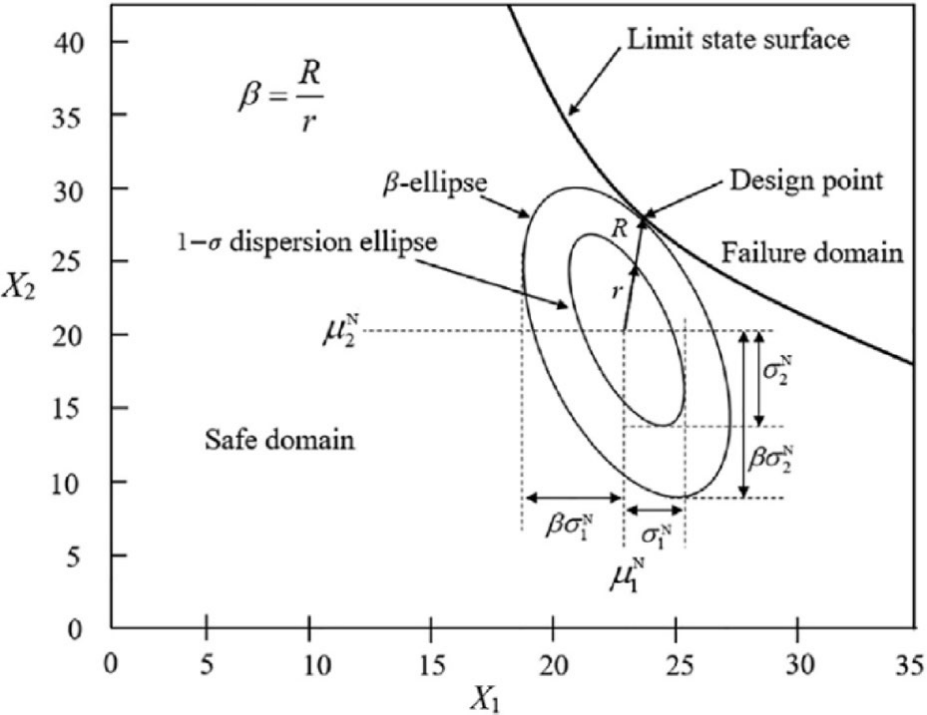
Design point and equivalent ellipses^29^.

In the spreadsheet-based implementation of the FORM algorithm, the design point is defined as the location on the limit state surface that delineates safe parameter combinations from unsafe ones. This point corresponds to the most probable set of parameters resulting in system failure^28^. In reliability analysis using FORM, uncertainties and the correlation structure among parameters are represented by a dispersion ellipse with a unit standard deviation (a one-standard-deviation ellipse) centered at the mean of the parameters (see Fig. 2). The safety margin is indicated by the reliability index, which corresponds to the shortest distance—in units of directional standard deviations (i.e., the ratio $\:R/r$)—from the mean point of the parameters to the most probable failure combination (the “design point”) on the limit state surface^29^.

Based on the reliability index, the probability of failure $\:{P}_{f}$ can be evaluated using Eq. (15)^22^:

15 $$\:{P}_{f}\approx\:1-\varphi\:\left(\beta\:\right)$$

In this expression, $\:{P}_{f}$ denotes the probability of failure, and $\:\varphi\:\left(\beta\:\right)$ represents the cumulative distribution function (CDF) of the standard normal variable.

A novel and efficient spreadsheet-based algorithm for the FORM approach has been proposed, which is expressed by Eqs. (16) and (17)^30^:

16 $$\:\beta\:=\underset{x\in\:F}{\mathrm{min}}\sqrt{{n}^{T}{R}^{-1}n}$$

17 $$\:{n}_{i}=\frac{{x}_{i}-{\mu\:}_{i}^{N}}{{\sigma\:}_{i}^{N}}={\varphi\:}^{-1}\left[F\left({x}_{i}\right)\right]$$

In this formulation, $\:n$ is a column vector consisting of the values $\:{n}_{i}$, which vary during the constrained optimization process; the corresponding value of $\:{n}_{i}$ is automatically computed. $\:F$ denotes the cumulative distribution function ($\:CDF$) of the non-normal variable at $\:{x}_{i}$, and $\:\varphi\:\left[\left({n}_{i}\right)\right]$ represents the cumulative distribution function of the standard normal variable $\:{n}_{i}$.

The reliability index $\:\beta\:$, as expressed in Eq. (16), can be computed using the FORM algorithm in conjunction with the built-in optimization tool “Solver” in Microsoft Excel. This process is carried out under the constraint of the limit state function $\:G\left(X\right)=0$, where the values of $\:{x}_{i}$ are calculated according to Eq. (16), and the values of $\:{n}_{i}$ are automatically adjusted during the optimization procedure.

### Correlation between safety factor and probability of failure

As explained in Sects. 3-1-3, evaluating the ground reaction curve and the support characteristic curve and determining the design safety factor requires solving Eq. (1) through (11). In this study, a new spreadsheet-based code was developed in Microsoft Excel, utilizing its Solver functionality, to compute these relations accurately. The computational workflow in this Excel spreadsheet proceeds as follows:

1. All parameters related to the mechanical and strength properties of the ground are defined as probabilistic variables with appropriate distributions, and the corresponding data are entered.
2. The intermediate computational parameters of the design are calculated according to Eqs. (1)–(4) and Eq. (6).
3. The values of elastic displacement and plastic displacement are computed using Eqs. (5) and (7), respectively.
4. The ground reaction curve is plotted on the internal force–displacement axes.
5. All parameters related to the mechanical and strength properties of the concrete support system are defined as probabilistic variables with appropriate distributions, and the relevant data are entered.
6. The maximum bearing capacity of the support system and its stiffness are calculated based on Eqs. (8) and (9).
7. The support characteristic curve is simultaneously plotted on the ground reaction curve diagram in the internal force–displacement space. In plotting this curve, the initial displacement of the support system ($\:{u}_{0}$​) must be determined in advance. To calculate $\:{u}_{0}$​, two limit values are first computed from Eqs. (18) and (19). If the value obtained from Eq. (18) is smaller than that from Eq. (19), then the result of Eq. (18) is taken as the initial displacement. Otherwise, the value from Eq. (19) is considered as the initial displacement of the concrete support system.

18 $$\:{u}_{0}=[1+exp{\left(\frac{-\frac{x}{R}}{1.1}\right)]}^{-1.7}{u}_{r}^{M}$$

19 $$\:{u}_{0}=1.3\:{u}_{r}^{el}$$

In this relation, $\:{u}_{r}^{M}$ represents the maximum ground displacement in the absence of a support system.

8. To calculate the intersection point of the ground reaction curve and the support characteristic curve, the displacement calculations of the ground in both the elastic and plastic ranges were reformulated based on the initial input parameters, eliminating the need for intermediate parameters (Eqs. (1)– (4) and (6)). Accordingly, Eq. (20) is defined as follows to compute the equilibrium pressure between the support system and the ground:

20 $$\:{u}_{r,G}^{pl}-{u}_{r,s}^{D}=0$$

In this relation, $\:{u}_{r,G}^{pl}$ represents the ground displacement at the equilibrium point, while $\:{u}_{r,s}^{D}$ denotes the support system displacement at equilibrium. Equation (20) can be reformulated in terms of the initial input parameters. Through this reformulation, the only unknown in the equation is the internal pressure $\:{p}_{i}$. By solving Eq. (20) implicitly using the Solver functionality in Microsoft Excel, the value of $\:{p}_{i}$ at the moment of ground–support equilibrium—equal to $\:{p}_{s}^{D}$—is obtained.

9. Now, using Eq. (11), the pressure-based design safety factor ($\:{SF}_{p}$​) can be calculated.

Since all input parameters related to the ground and the support system follow probabilistic distributions, a macro was implemented in the designed spreadsheet for computing the CCM-based safety factor. The macro generates input data in appropriate quantities according to the specified mean, standard deviation, and distribution type, and for each execution it applies Steps 1–10 to a unique dataset in which all input variables are sampled simultaneously in a probabilistic manner. The workflow of the macro proceeds as follows:

- For all input parameters of the ground and the concrete lining, it generates a unique sample consistent with the specified distribution type, mean, standard deviation, etc.
- It simultaneously feeds the generated inputs into the computation sheet and performs all calculations.
- In a dedicated worksheet for inputs and results, it stores—on a new row with appropriate headers—all input data associated with that computation run.
- It calls Excel’s Solver to compute $\:{p}_{s}^{D}$​ and the pressure-based safety factor ($\:{SF}_{p}$​).
- All output data and results—including $\:{p}_{s}^{D}$, equilibrium displacement, maximum bearing capacity of the support system, support system stiffness, safety factor, maximum ground displacement, and others—are stored in the “Input Parameters and Results” sheet, appended to the same row of the corresponding input set, with appropriate labels.
- Steps 1 through 5 are repeated as many times as specified by the user to generate input datasets according to the defined probabilistic distributions. Consequently, the storage sheet for inputs and results will ultimately contain as many unique rows as the user requires, each row holding both the probabilistic input data (simultaneously generated for all variables) and the corresponding outputs.
- The mean and standard deviation of the output parameters are computed, and the corresponding plots are generated.
- Finally, by simultaneously considering the probabilistic distributions of all input parameters, the percentage probability of failure ($\:{P}_{f}$) is explicitly and accurately calculated. To determine the probability of failure, the proportion of cases where the safety factor falls below unity is computed relative to the total number of cases.

Recent developments in AI-assisted and explainable modeling have further expanded the role of data-driven tools in civil and geotechnical engineering. For example, interpretable machine-learning frameworks have been successfully applied to structural and material behavior prediction, demonstrating how transparent AI models can support engineering decision-making^31^. Similarly, advanced AI-based formulations have been used to model the mechanical performance of cementitious composites with high accuracy and interpretability^32^. These studies highlight the growing relevance of explainable AI in engineering applications and support the motivation for integrating an explicit, AI-derived surrogate equation within the proposed reliability-based tunnel lining framework.

## Derivation of the surrogate model using gene expression programming

To derive an explicit and interpretable surrogate model for predicting the pressure-regulated safety factor ($\:{SF}_{p}$), Gene Expression Programming (GEP) was employed as the symbolic regression framework. GEP is an evolutionary computation technique that combines the global search capability of Genetic Algorithms with the structured expression-tree representation of Genetic Programming. Unlike black-box machine learning approaches, GEP evolves closed-form analytical expressions, thereby offering both predictive capability and direct engineering interpretability. This characteristic is particularly advantageous in geomechanical systems, where nonlinear interactions among rock mass properties, in-situ stresses, and support characteristics govern stability behavior, yet explicit analytical relationships are generally unknown.

The dataset used for surrogate modeling was generated from the probabilistic Convergence–Confinement Method (CCM) computational framework. In total, 1000 Monte Carlo-simulated records were produced for each of the nine parameter combinations, resulting in a dataset that captures a wide range of physically consistent tunnel–support configurations derived from systematic parametric simulations. The predictor variables incorporated into the GEP model were the principal mechanical and geometrical parameters influencing tunnel stability: tunnel radius ($\:R$), in-situ stress ($\:{\sigma}_{0}$), intact rock compressive strength ($\:{\sigma\:}_{ci}$), Geological Strength Index ($\:GSI$), Hoek–Brown material constant ($\:{m}_{i}$), lining elastic modulus ($\:{E}_{c}$), lining compressive strength ($\:{\sigma\:}_{cc}$), and lining thickness ($\:{t}_{c}$). These parameters form the terminal set of the symbolic regression model and directly correspond to the governing variables within the CCM formulation. The response variable was the pressure-controlled safety factor ($\:{SF}_{p}$).

Prior to model development, the dataset was randomly shuffled to remove ordering bias. The data were then partitioned into two mutually exclusive subsets: 80% for training and 20% for independent testing. The training subset was used exclusively during evolutionary optimization, while the testing subset remained completely unseen until final validation. This strict separation ensured elimination of data leakage and enabled an unbiased evaluation of predictive generalization.

To enhance numerical stability and ensure balanced evolutionary search, the predictor variables were standardized using Z-score normalization:

21 $$\:{X}_{norm}=\frac{X-\mu\:}{\sigma\:}$$

where $\:\mu\:$ and $\:\sigma\:$ denote the mean and standard deviation computed solely from the training dataset. The same parameters were subsequently applied to the testing dataset to preserve consistency. The target variable $\:{SF}_{p}$ was retained in its physical scale to maintain interpretability of the evolved analytical expression.

The GEP implementation was conducted in a Python-based computational environment integrating the GEPPY library with the DEAP evolutionary framework. To guarantee strict reproducibility of the stochastic evolutionary process, deterministic execution was ensured by fixing the pseudo-random seeds for both the Python random module and NumPy. Because evolutionary optimization relies on stochastic operations such as mutation and crossover, seed control ensures identical results upon repeated execution under identical settings.

Individuals were encoded as multi-gene chromosomes using the GeneDc architecture. Each chromosome consisted of multiple genes, each representing a symbolic sub-expression that is later translated into an expression tree. These genes were linked via an additive linking function, resulting in a composite nonlinear analytical model. This multi-gene structure enhances representational flexibility and allows complex nonlinear relationships to be decomposed into smaller symbolic components. Random Numerical Constants (RNCs) were incorporated within a predefined range to enable adaptive coefficient optimization during the evolutionary process.

The function set was deliberately restricted to fundamental arithmetic operators: addition, subtraction, multiplication, and protected division. The protected division operator prevents undefined numerical behavior by safely handling potential division-by-zero events during evolution. This constrained function set preserves mathematical robustness and physical interpretability while avoiding unnecessary functional complexity.

Model performance during evolution was evaluated using the Root Mean Square Error (RMSE) between predicted and numerical $\:{SF}_{p}$ values. The optimization objective was explicitly defined as minimization of RMSE, thereby directing evolutionary pressure toward increasingly accurate solutions. Invalid or numerically unstable expressions were automatically penalized through safeguarded operator implementations within GEPPY.

The evolutionary search was governed by well-defined hyperparameters to balance exploration and exploitation. The population size was set to 50 individuals and evolution proceeded for 50 generations. Tournament selection with a tournament size of three was used for parent selection, providing moderate selection pressure while maintaining diversity. Elitism preserved the best-performing individual in each generation, enhancing convergence stability. A Hall-of-Fame mechanism retained the top three individuals discovered throughout the evolutionary process, ensuring preservation of the globally best expression.

A diverse suite of genetic operators was employed to enrich the search process, including uniform mutation, inversion, insertion sequence (IS) transposition, root insertion sequence (RIS) transposition, gene transposition, Dc-specific mutation, RNC array mutation, and one-point, two-point, and gene-level crossover. Operator probabilities were explicitly defined within the GEPPY toolbox configuration. This diversity of operators promotes both local refinement and structural exploration, thereby reducing the likelihood of premature convergence.

Overfitting was mitigated through multiple complementary mechanisms. First, strict separation of training and testing datasets prevented information leakage. Second, chromosome complexity was controlled by fixing gene count and head length, limiting structural bloat. Third, moderate population and generation sizes reduced the risk of over-specialization. Fourth, performance consistency between training and testing datasets was carefully monitored to verify generalization capability. The close agreement between training and testing metrics confirmed that the model captured underlying physical trends rather than memorizing training data.

Upon completion of evolutionary optimization, the best-performing chromosome stored in the Hall-of-Fame was translated into executable mathematical form using the compile_ function of GEPPY. However, as commonly observed in GEP, the raw evolved expression may contain redundant operations, nested algebraic structures, neutral elements (such as multiplication by one or addition of zero), or structurally equivalent yet unnecessarily complex sub-expressions. Therefore, a rigorous two-stage symbolic simplification procedure was implemented to ensure mathematical compactness, interpretability, and engineering usability of the final predictive equation.

In the first stage, structural simplification was performed using GEPPY’s built-in simplify() function:

22 $$\:symplified\_best\hspace{0.17em}=\hspace{0.17em}gep.simplify(best\_ind)$$

Because GEP chromosomes are encoded as linear genomes and expressed as expression trees, evolutionary operations may produce algebraically correct but structurally bloated expressions. The GEPPY simplifier removes syntactic redundancies introduced by mutation, inversion, and transposition, prunes inactive genes, eliminates neutral subtrees, and compresses trivial operator sequences. This process reduces expression depth and structural complexity without altering predictive functionality, thereby improving numerical stability and clarity.

In the second stage, the resulting expression was subjected to algebraic reduction using the SymPy symbolic mathematics engine. The simplified expression was converted into symbolic form and processed through SymPy’s manipulation routines, including factorization, expansion, collection of like terms, and constant folding. This phase ensures combination of equivalent terms, elimination of algebraic redundancies, reduction of nested arithmetic structures, and generation of a canonical symbolic representation.

When linear scaling was enabled, the final model was expressed in affine form:

23 $$\:S{F}_{p}=a\cdot\:f(R,{\sigma\:}_{0},{\sigma\:}_{ci},GSI,{m}_{i},{E}_{c},{\sigma\:}_{cc},{t}_{c})+b$$

where $\:f(\cdot\:)$ represents the evolved symbolic structure and $\:a$ and $\:b$ are optimized scaling coefficients embedded within the chromosome. Linear scaling enhances regression accuracy while preserving structural interpretability.

The combined GEPPY–SymPy simplification pipeline provides several important advantages. First, it improves interpretability by producing a concise and physically meaningful analytical expression. Second, it reduces computational expense by lowering the number of arithmetic operations required during prediction. Third, it enhances numerical robustness by eliminating unnecessary nested computations that could amplify floating-point errors. Fourth, it facilitates analytical sensitivity analysis and parametric investigation because the final expression is compact and differentiable.

Importantly, the simplification process preserves strict functional equivalence; it reduces structural complexity without modifying predictive behavior. Consequently, the final reported equation (Eq. 24) represents the most parsimonious symbolic form discovered through evolutionary search, achieving a balance among predictive accuracy, mathematical elegance, and engineering practicality.

24 $$\:{SF}_{p}=\:-3.15+0.13\:\frac{R\:{m}_{i}\:\left({E}_{c}+{\sigma\:}_{0}{\:\sigma\:}_{cc}\right)+{\sigma\:}_{cc}\:\left[R\:\left({E}_{c}+{\:\sigma\:}_{ci}+GSI\right)+{\:t}_{c}\right]}{R\:({E}_{c}+{\sigma\:}_{0}{\:\sigma\:}_{cc})}$$

The predictive capability of the final surrogate model was evaluated using the independent testing dataset. Performance metrics included the coefficient of determination ($\:R^2$), Root Mean Square Error (RMSE), Mean Absolute Error (MAE), and prediction bias. Residual analysis confirmed the absence of systematic deviation and demonstrated strong agreement between surrogate predictions and probabilistic CCM results.

**Fig. 3:**
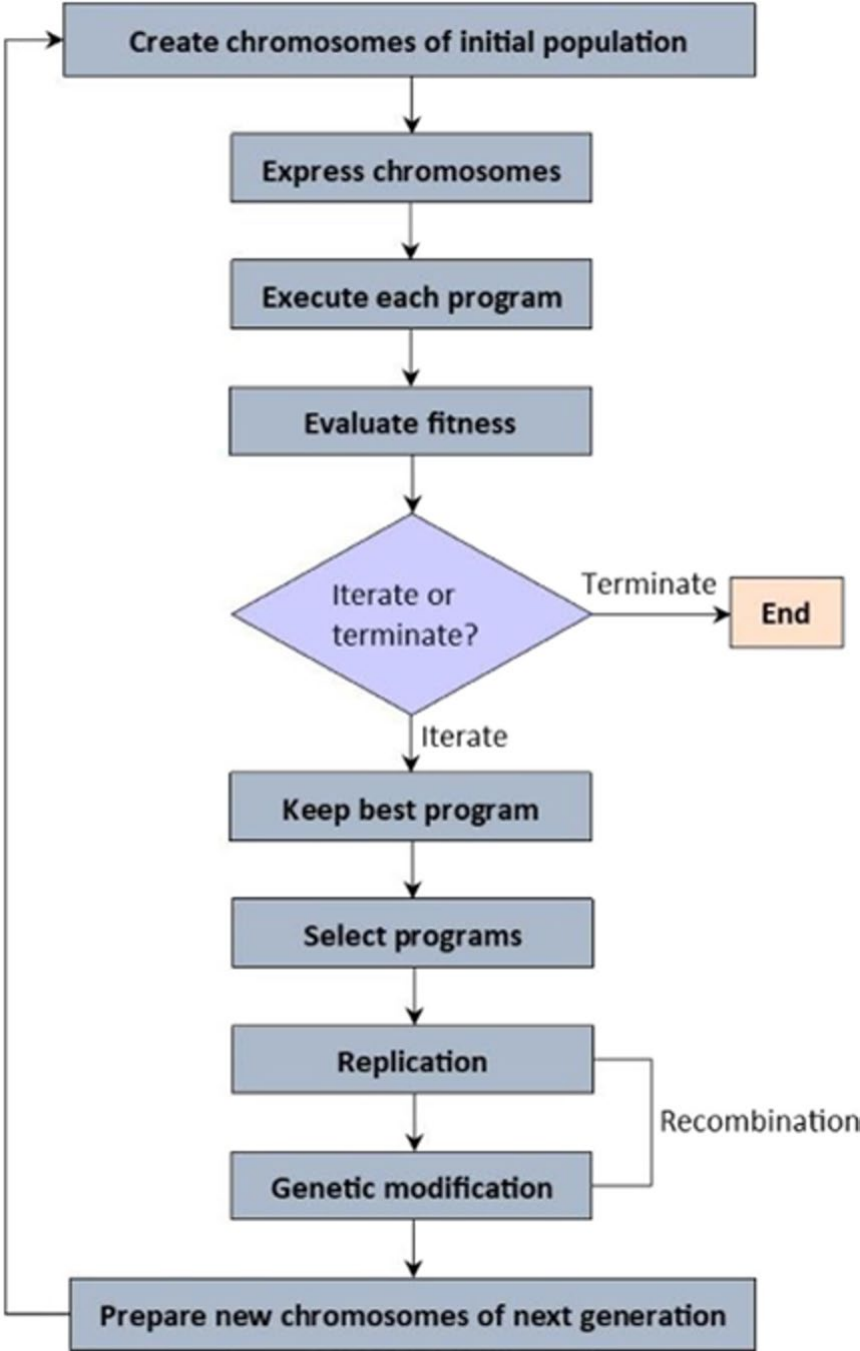
GEP flowchart.

For complete transparency and reproducibility, all datasets, algorithmic hyperparameters, and source codes used in the GEP modeling process are publicly available through the repository specified in the Data Availability Statement. Deterministic seed control ensures exact regeneration of the reported surrogate model. Figure 3 presents a comprehensive flowchart summarizing the full workflow, including dataset generation, preprocessing and normalization, evolutionary optimization, symbolic simplification, and independent validation.

## Input data

One of the objectives of this study is to present a novel approach for evaluating the safety factor based on reliability and for calculating the probability of failure of a tunnel excavated in weak rock and stabilized with concrete lining. As previously described, the convergence–confinement method was adopted in this study based on the work of Carranza-Torres. The input parameters for Tunnel, ground and concrete lining conditions were treated as probabilistic variables, as summarized in Table 1.

In this study, weak rock conditions are represented using a Normal distribution of GSI with a mean of 25 and a standard deviation of 5, which corresponds to the “poor” to “very poor” rock mass classes commonly associated with weak ground conditions.

**Table 1:** Input Ground and Support Parameters and Probabilistic Distribution Characteristics.

Parameter	Symbol	Distribution Type	Unit	Mean	Standard Deviation
Tunnel excavation radius	R	Normal	m	4.5	0.5
In-situ stress	$\:{\sigma\:}_{o}$	Normal	MPa	1.5	0.4
Uniaxial compressive strength of rock material	$\:{\sigma\:}_{ci}$	Normal	MPa	15	3.5
Hoek–Brown rock constant	$\:{m}_{i}$	Normal	-	5	1
Geological Strength Index (GSI)	$\:GSI$	Normal	-	25	5
Uniaxial compressive strength of concrete lining-Conventional	$\:{{\upsigma\:}}_{cc}$	Normal	MPa	20	2
Uniaxial compressive strength of concrete lining-FRPC	$\:{{\upsigma\:}}_{cc}$	Normal	MPa	65	6.5
Elastic modulus of concrete lining	$\:{E}_{c}$	Normal	GPa	20	2
Thickness of concrete lining	$\:{t}_{c}$	Normal	mm	10 to 300	1 to 30 respectively

## Results

In this study, with the objective of analyzing the safety of tunnel lining under varying geological conditions, a series of comprehensive numerical and statistical analyses were carried out based on the mechanical properties of the rock mass and the concrete lining. In the first stage, the modeling inputs—including geomechanical variables such as initial vertical stress ($\:{\sigma}_{0}$), uniaxial compressive strength of intact rock ($\:{\sigma\:}_{ci}$), parameter $\:m$, Geological Strength Index ($\:GSI$), and tunnel excavation radius ($\:R$)—were defined probabilistically from among the full set of ground parameters. Subsequently, the properties of the lining material, including compressive strength of the concrete lining ($\:{\sigma\:}_{cc}$), elastic modulus ($\:{E}_{c}$), and lining thickness ($\:{t}_{c}$), were also introduced as design variables with probabilistic distributions.

The analyses were conducted under two scenarios of uniaxial compressive strength for the concrete lining, 20 MPa and 65 MPa, and for a range of practical concrete thicknesses between 10 and 300 mm. Three different analytical approaches were employed to evaluate the performance of the support system: (1) deterministic analysis based on the mean values of the input parameters, (2) probabilistic analysis accounting for uncertainty in all input parameters, and (3) a machine learning–based model trained on the generated datasets to predict the safety factor under varying conditions.

In this study, the reliability analysis is formulated using the pressure-based safety factor ($\:S{F}_{p}$), which reflects compression-controlled failure of the lining. While $\:S{F}_{p}$ is adopted as the sole performance function in the current framework, the methodology can be extended to displacement-based or strain-rate-based criteria by redefining the limit-state function and updating the CCM formulation accordingly.

Accordingly, for each combination of lining strength and thickness, outputs such as the deterministic safety factor ($\:{SF}_{p}\_det$), the probabilistic safety factor ($\:{SF}_{p}\_prob$), the safety factor predicted by the artificial intelligence model ($\:{SF}_{p}\_AI$), the reliability index ($\:\beta\:$), and the probability of failure ($\:{P}_{f}$) were obtained using the FORM-based approach and the Excel Solver algorithm. The discrepancies between the AI method and the other approaches were also examined and analyzed through relevant statistical indices.

The results not only enabled comparison across different methods but also clearly illustrated the dependence of system performance on design parameters. For example, with increasing lining thickness, the safety factor consistently increased across all methods, although the magnitude of this change depended on the compressive strength of the lining. Moreover, in probabilistic approaches and AI-based models, the trend of safety factor variations exhibited greater complexity, arising from the nonlinear interactions among input parameters and their statistical characteristics. The findings also revealed that machine learning models, particularly at intermediate thicknesses, provide satisfactory accuracy in safety estimation while significantly accelerating the analysis process.

### Effect of lining thickness and concrete strength on the design safety factor

**Fig. 4:**
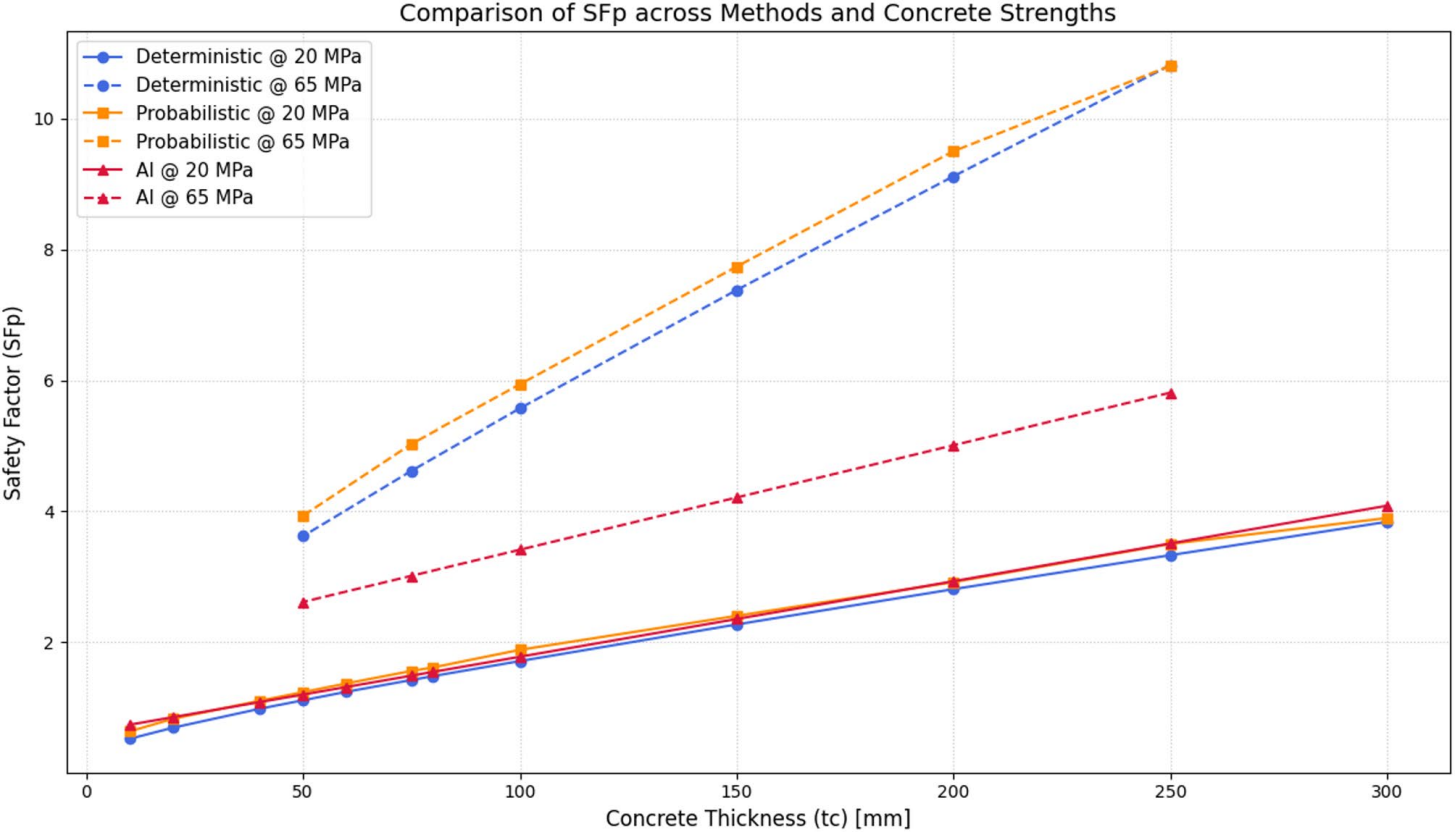
Comparison of the safety factor ($\:{SF}_{p}$) in deterministic, probabilistic, and artificial intelligence methods for different thicknesses and strengths of Concrete.

In Fig. 4, the variation of the safety factor ($\:{SF}_{p}$) with respect to concrete lining thickness ($\:{t}_{c}$) is compared for two compressive strengths, 20 MPa and 65 MPa, using three approaches: deterministic, probabilistic, and artificial intelligence (AI). As observed, increasing the lining thickness leads to higher safety factor values across all methods and for both strength levels, reflecting improved stability of the support system under reinforced conditions.

When comparing the methods, the probabilistic analysis yielded the highest $\:{SF}_{p}$ values at most thicknesses, followed by the deterministic approach. In contrast, the AI-based model predicted more conservative values, particularly for the lower strength case ($\:{\sigma\:}_{c}$ = 20 MPa), where its deviation from classical methods was more pronounced. Moreover, the differences among the methods were relatively smaller at 65 MPa strength, which may be attributed to the convergence of model predictions under stronger material conditions.

These findings indicate that machine learning models tend to provide more cautious safety factor predictions, especially under weaker material conditions or less stable scenarios, which can be considered an advantage in safety-oriented design.

### Effect of lining thickness and concrete strength on the probability of failure

**Fig. 5:**
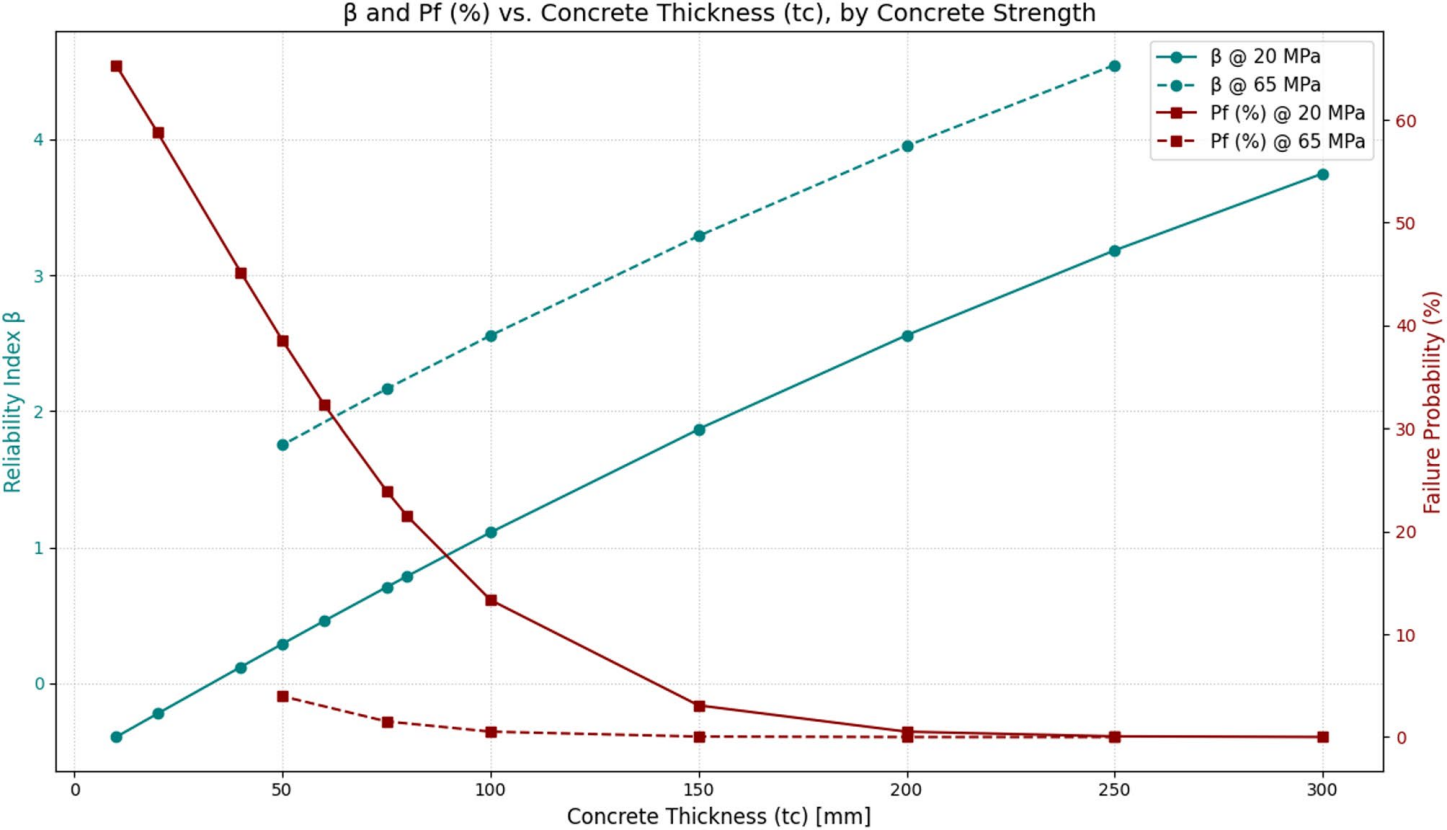
Relationship between the reliability index ($\:\beta\:$\beta) and failure probability ($\:{P}_{f}$) with concrete thickness at two different compressive strengths.

Figure 5 illustrates the variation of the reliability index ($\:\beta\:$) and failure probability ($\:{P}_{f}\%$) with concrete lining thickness ($\:{t}_{c}$) for two uniaxial compressive strengths, 20 MPa and 65 MPa, plotted simultaneously using dual vertical axes. As expected, with increasing lining thickness, the value of $\:\beta\:$ consistently rises while the probability of failure decreases. This trend is evident for both concrete strengths, though the rate of change is more pronounced at the lower strength.

Specifically, for the 20 MPa case, the reliability index $\:\beta\:$ drops to negative or very low values at thicknesses below 50 mm, indicating a high probability of failure. By contrast, the higher strength ($\:{\sigma\:}_{c}$=65 MPa) provides considerable stability to the support system, with $\:\beta\:$ reaching safer values even at relatively small thicknesses. In this case, the probability of failure rapidly decreases to below 1%, whereas for weaker concrete this reduction is only achieved at larger thicknesses.

This analysis highlights that increasing thickness alone does not guarantee stability; the mechanical quality of the material—particularly the compressive strength of the concrete—plays a decisive role in the safety of the support system.

**Fig. 6:**
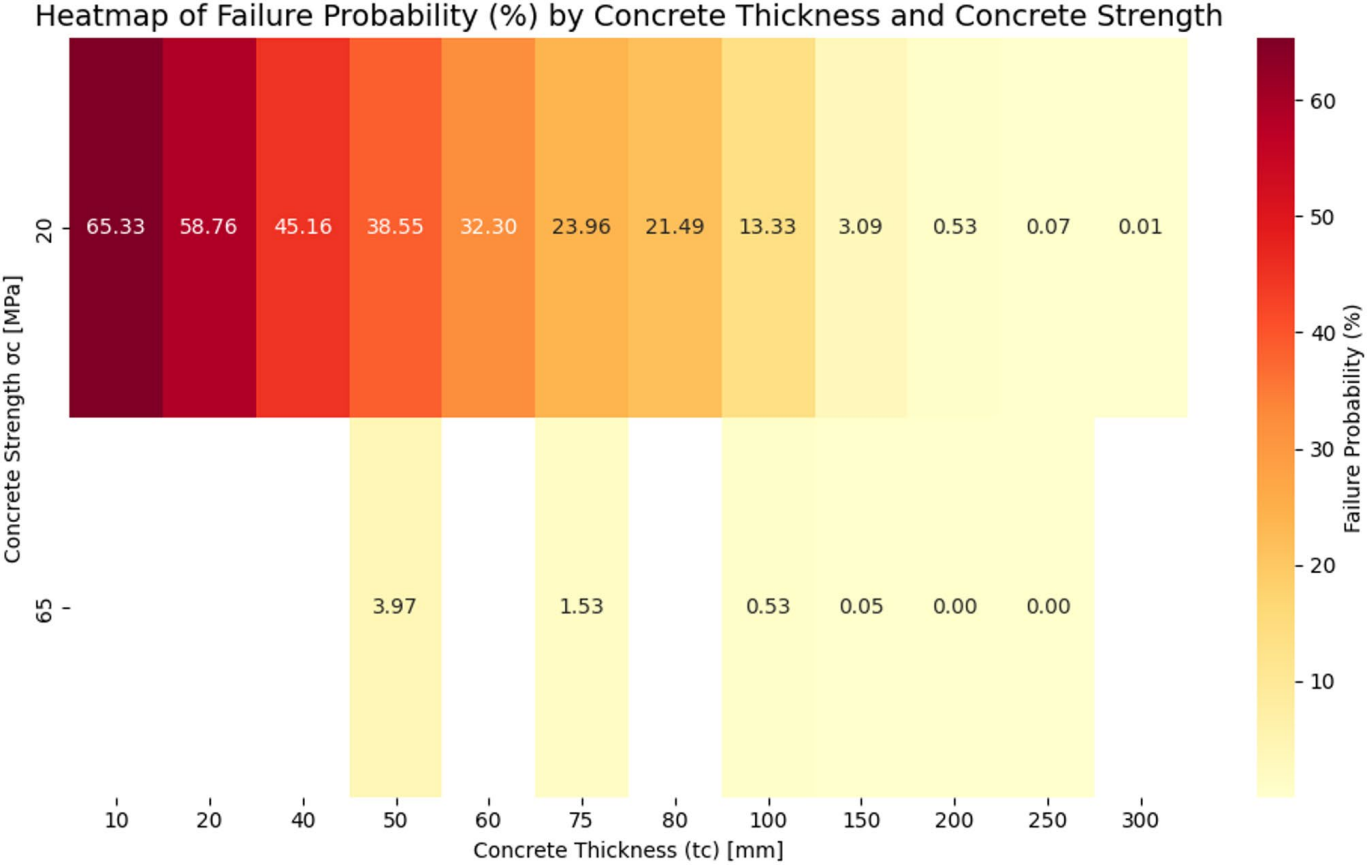
Heat map of failure probability as a function of concrete thickness and compressive strength.

Figure 6 presents a heat map of the failure probability ($\:{P}_{f}\%$) of the support system as a function of concrete thickness ($\:{t}_{c}$) and compressive strength ($\:{\sigma\:}_{c}$). The diagram is provided for two commonly used compressive strengths in design, 20 MPa and 65 MPa. It is observed that at the lower strength (20 MPa), the probability of failure is very high at small thicknesses, exceeding 65%. With increasing thickness, a continuous and significant reduction in failure probability occurs, reaching negligible values (close to zero) at thicknesses greater than 200 mm.

In contrast, for a compressive strength of 65 MPa, the probability of failure is already very low—below 2%—at intermediate thicknesses such as 75 mm, and is entirely eliminated at higher thicknesses. This behavior confirms the critical role of concrete compressive strength in improving support system safety. In other words, if increasing thickness is limited due to construction or economic constraints, enhancing material quality (increasing $\:{\sigma\:}_{c}$) can serve as an effective strategy for risk reduction. Moreover, the trends depicted in this diagram suggest the presence of an optimal balance between thickness and strength, which should be considered in the optimal design of tunnel linings.

### Evaluation of the accuracy of the machine learning model compared to probabilistic methods in determining $\:{\boldsymbol{S}\boldsymbol{F}}_{\boldsymbol{p}}$

**Fig. 7:**
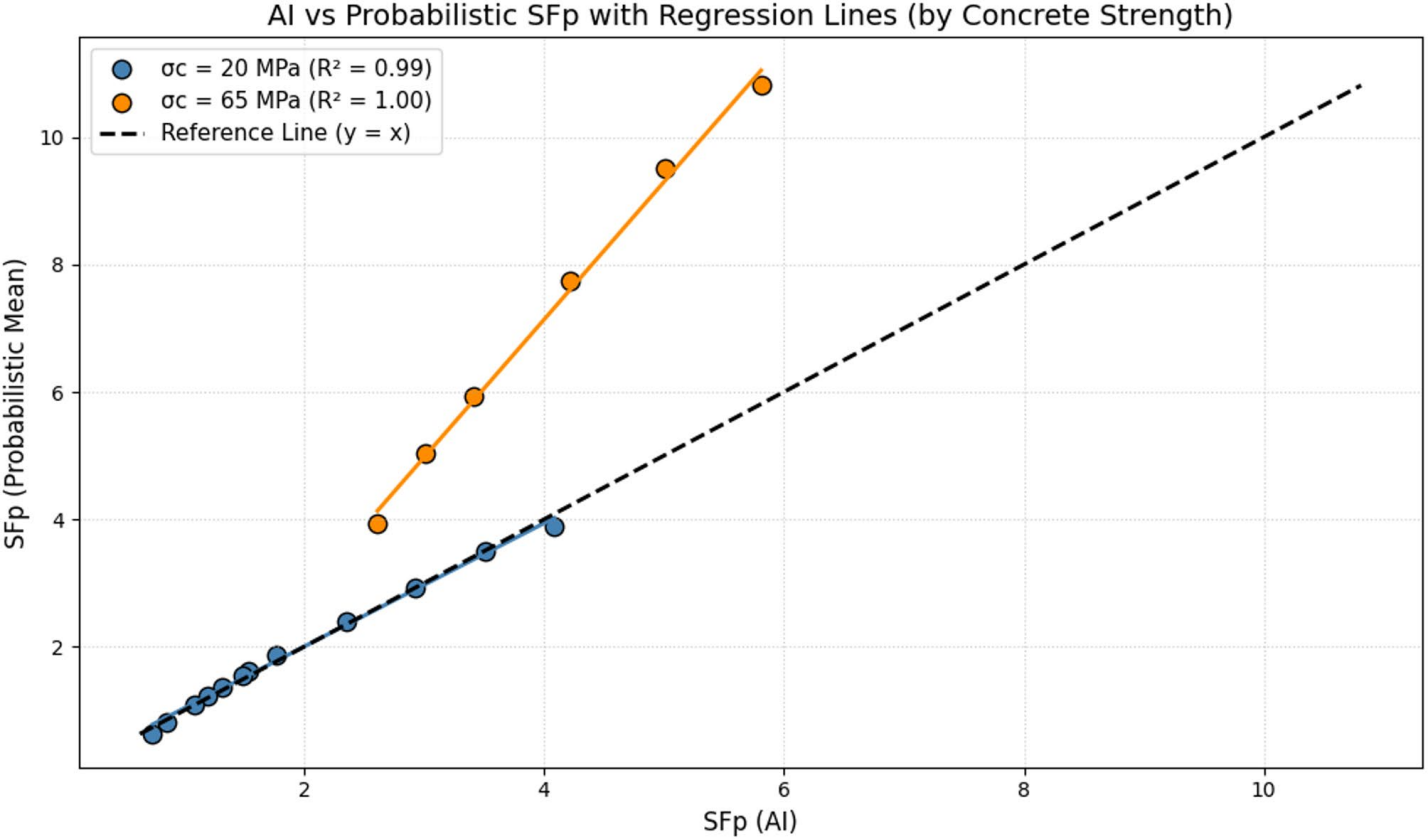
Correlation analysis between the safety factor predicted by artificial intelligence and the probabilistic mean analysis at different strength levels.

Figure 7 provides a direct comparison between the outputs of the artificial intelligence model and the mean safety factor obtained from probabilistic analysis for two compressive strengths of concrete lining, 20 MPa and 65 MPa. In this figure, a dataset is plotted for each strength together with the corresponding regression line, allowing for a visual assessment of the consistency between the two methods. The results show that for both strength levels, the data points lie consistently along a clear linear trend, demonstrating the strong capability of the AI model to reproduce the outcomes of probabilistic analysis.

The position of the data points relative to the reference line x = y is particularly noteworthy. As shown, the majority of the points fall below this line, indicating that the values predicted by the AI model are relatively lower than the mean values from probabilistic analysis. This behavior can be interpreted as a conservative tendency, since the AI model effectively proposes lower safety factors, thereby enhancing the safety margin in design. While probabilistic analysis provides more precise values that fully reflect the uncertainty distributions, the AI model simplifies the process and produces slightly more conservative values, making it a practical tool when minimizing the risk of failure is a priority.

The differences between the two compressive strengths are also significant. For the lower strength case (20 MPa), the range of calculated safety factors is narrower. Although the AI and probabilistic results align closely, a slight deviation in favor of the AI’s conservative predictions remains evident. In contrast, for the higher strength case (65 MPa), the range of safety factor variation is wider, with higher $\:{SF}_{p}$ values recorded. Even in this scenario, the data exhibit a clear linear trend, and the AI model continues to predict lower values than the probabilistic mean, yet with the same degree of accuracy and consistency.

From a practical standpoint, these findings are highly significant. Although probabilistic analysis is an accurate and reliable method, it requires heavy and time-consuming computations, making it costly to apply across all design scenarios. In contrast, the AI model, trained on prior datasets, offers rapid and relatively conservative predictions. As such, in underground engineering projects with limited time and resources, this model can serve as an alternative or complementary tool to classical probabilistic methods.

Overall, Fig. 7 demonstrates that the AI model employed in this study not only has the ability to accurately reproduce analytical trends but also exhibits a conservative bias, making it a valuable option for safety-based design under uncertainty and in complex geological environments.

### Comparison between FORM and proposed framework

In this study, the effect of concrete thickness and compressive strength on the failure probability of the support system was evaluated using two different analytical approaches. The first approach was based on numerical solutions through Excel Solver, while the second employed the FORM method within the framework of reliability analysis. The evaluations were conducted for two types of concrete with characteristic compressive strengths of 20 MPa and 65 MPa, and the results were presented in charts showing the variation of failure probability with respect to concrete thickness.

For concrete with a compressive strength of 20 MPa, it was observed that at small thicknesses (below 50 mm), the probability of failure is significant. Specifically, the RT software reported values of about 65%, while Solver gave approximately 75%. As thickness increases, the probability of failure decreases in both methods. At thicknesses between 100 and 150 mm, both methods yield a failure probability below 15%, and with further increases up to around 200 mm, the values approach zero. This relative convergence at higher thicknesses highlights the strong effect of increasing thickness on improving the structural performance of concrete at lower strength levels. However, the slight difference in the rate of reduction between the two methods indicates that FORM, by incorporating statistical variability and nonlinear material behavior, is capable of modeling the actual structural response with greater accuracy.

In contrast, the analysis for concrete with a compressive strength of 65 MPa indicated a far more stable performance against failure. Even at smaller thicknesses (around 50 mm), the failure probability was only about 4% according to FORM and less than 0.5% with Solver. With increasing thickness, both methods quickly reported probabilities approaching zero. In this case, the difference between the two methods became more evident: Solver considered the probability of failure to be zero across all thicknesses, whereas FORM retained small fluctuations. This demonstrates that when the system’s safety is inherently high due to strong material properties, simpler models such as Solver may present overly optimistic results and therefore may not be fully reliable for safe design purposes.

The comparison between the two charts also clearly shows that failure probability is far more sensitive to thickness in lower-strength concrete than in higher-strength concrete. In other words, increasing thickness plays a much more critical role in risk reduction for 20 MPa concrete, making reliability-based analysis particularly important in such cases. For higher-strength concrete, however, optimal design should focus more on economic, constructional, and crack-control considerations rather than initial stability.

**Fig. 8:**
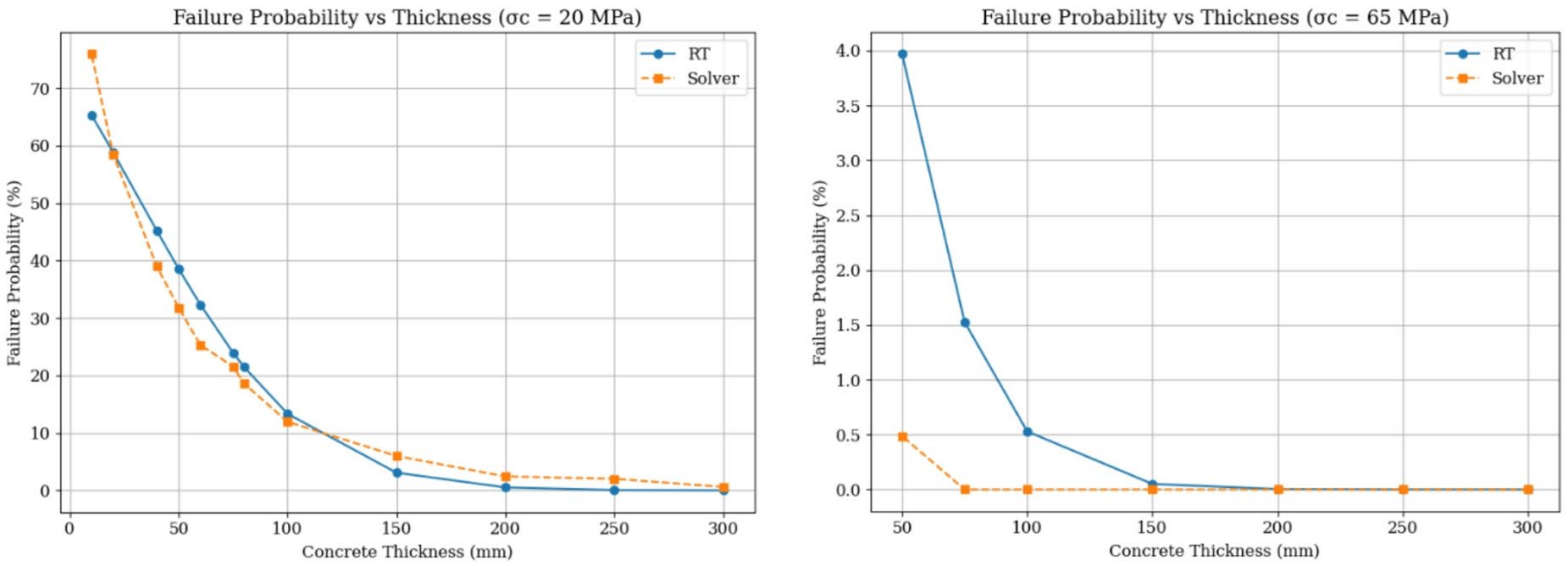
Comparison between the results of the FORM method and Proposed Framework.

Overall, these analyses demonstrate that the selection of concrete thickness must simultaneously account for material compressive strength and the level of statistical analysis employed. The use of advanced reliability methods such as FORM allows for a more accurate evaluation of system performance under uncertainty, whereas reliance solely on traditional deterministic analyses can lead to inaccurate and non-conservative estimates under critical conditions. Therefore, integrating rigorous statistical analyses with engineering design considerations plays a crucial role in enhancing both the safety and efficiency of underground support systems (Fig. 8).

### Relationship between probability of failure and safety factor (SF)

In this section, the relationship between deterministic analysis results and probabilistic outcomes obtained from the FORM method is examined. The deterministic safety factor ($\:{SF}_{p}$) values were calculated by solving the equilibrium equations in Excel using the Solver tool, and the results were compared with the failure probabilities ($\:{P}_{f}\%$) derived from the FORM approach.

The data analysis reveals a nonlinear and inverse relationship between $\:{SF}_{p}$ and $\:{P}_{f}\%$. Specifically, as $\:{SF}_{p}$ decreases from about 12 to values below 1, the probability of failure increases continuously, reaching levels above 65% under certain conditions. This trend clearly demonstrates that as system stability decreases from a deterministic perspective, the probabilistic likelihood of failure correspondingly rises. The correlation indicates that while deterministic analysis alone may not be sufficient for a full safety evaluation, it often reflects the overall behavioral trend of the system.

**Fig. 9:**
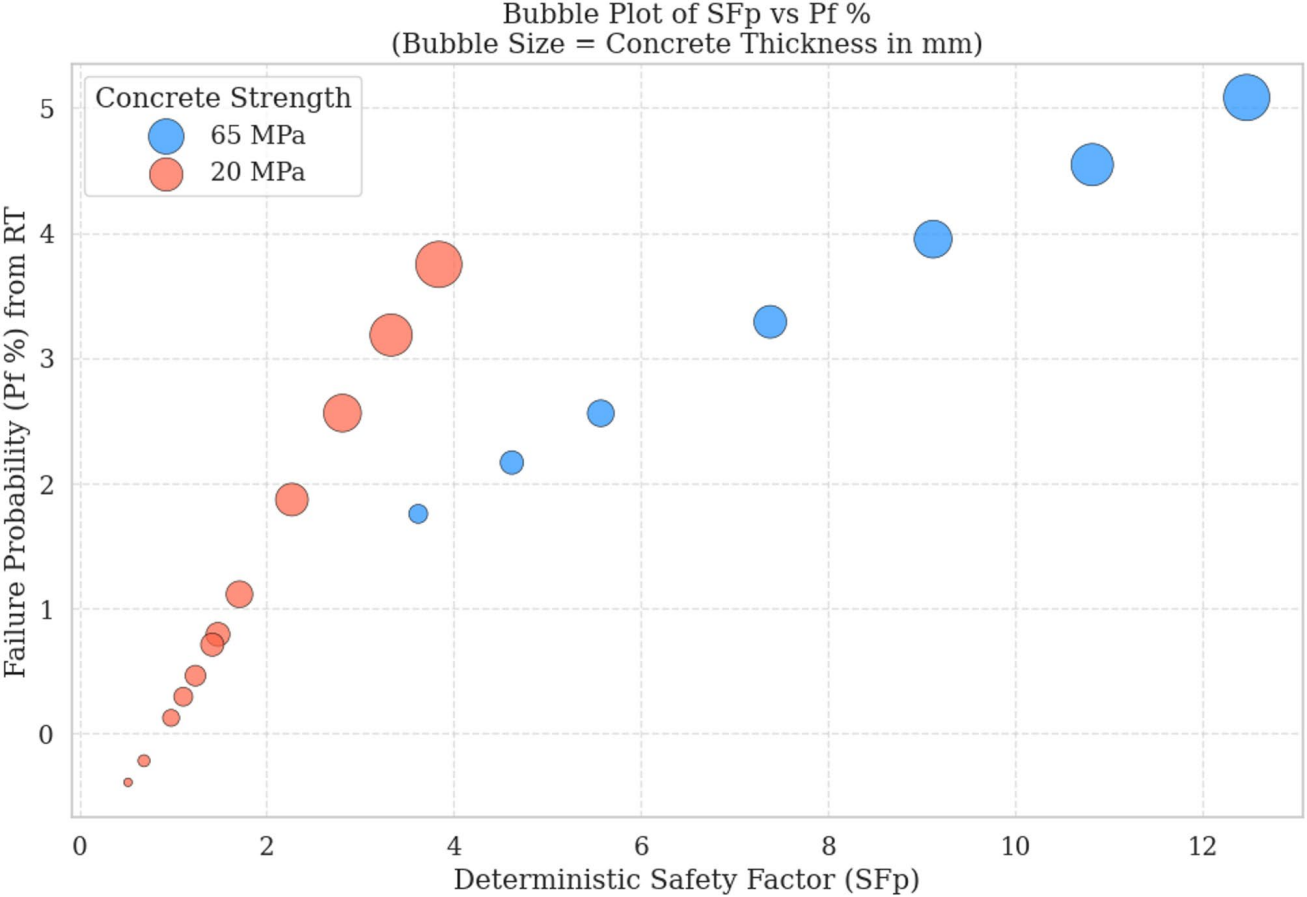
Comparison of the relationship between probability of failure and safety factor (SF).

As shown in Fig. 9, in values of $\:{SF}_{p}$ near 1 (i.e., the critical range), $\:{P}_{f}\%$ increases sharply and abruptly. For example, when $\:{SF}_{p}$ was reported as 1.11, the probability of failure was estimated at more than 38%, and at $\:{SF}_{p}=0.52$, it rose beyond 65%. This behavior clearly reflects the high sensitivity of the system near the stability boundary, where FORM, by incorporating statistical fluctuations, provides a more accurate estimation of failure probability. Moreover, FORM was able to predict significant probabilities of failure (up to about 15%) even when $\:{SF}_{p}$ remained relatively high (e.g., 1.42 or even 1.71), highlighting that deterministic analysis via Solver may overestimate safety in some cases.

The structured correlation between $\:{SF}_{p}$ and $\:{P}_{f}\%$ suggests a strong potential for developing regression models or machine learning algorithms based on these relationships. The observed trend, especially in critical regions, offers opportunities to derive predictive analytical relations that could be applied in rapid design or engineering decision-making contexts.

The relationship between deterministic safety factors from proposed framework and probabilistic failure probabilities from FORM confirms that reductions in $\:{SF}_{p}$ are accompanied by sharp increases in failure probability, particularly in the critical zone. While $\:{SF}_{p}$ provides preliminary insights into system stability, probabilistic analysis via FORM allows for a more precise assessment of risk. Therefore, it is recommended that both deterministic and probabilistic approaches be applied in a complementary manner, particularly for safety-critical designs.

## Conclusion

In this work, we proposed a reliability-based framework for the design of tunnel linings in weak rock, combining the convergence–confinement method with probabilistic analysis and a surrogate AI model. It is acknowledged that the present analysis adopts the simplifying assumptions of a hydrostatic in situ stress field, circular tunnel geometry, and zero dilation angle, consistent with the classical CCM formulation. By treating both rock mass and lining properties as random variables, the framework enables unified evaluation of the pressure-controlled safety factor, reliability indices, and failure probabilities. The explicit formulation of the CCM equations, combined with the use of Excel Solver, provides a transparent and reproducible means of locating the ground–support equilibrium without reliance on intermediate assumptions. In the current study, the framework is formulated solely for the pressure-controlled performance function. While other definitions—such as displacement-based or strain-rate-based safety factors—are not incorporated here, they may be developed in future work by redefining the limit-state condition accordingly.

Several insights emerged from the study. First, the influence of lining thickness and compressive strength is not only significant but also directional: thicker linings consistently improve stability, while higher-strength concretes reduce the sensitivity of designs to small thickness changes. Second, the comparison between deterministic estimates and full probabilistic analysis revealed that simplified approaches can underestimate risk, particularly in marginal conditions. This underscores the value of reliability indices as a complement to traditional safety factors. Third, the surrogate machine learning model reproduced the probabilistic trends with high fidelity, offering a practical tool for rapid parametric studies, though care is needed near boundary conditions. Finally, the development of fiber-reinforced reactive powder concrete demonstrated that material innovation can meaningfully shift reliability outcomes, providing a viable option when construction constraints limit thickness.

Beyond these technical findings, the broader contribution of this study lies in reframing tunnel lining design from a fixed-thickness prescription to an optimization problem guided by reliability targets. This shift encourages designers to balance thickness and material quality in a way that is both economical and resilient to field variability. While further validation under diverse geological settings would strengthen the generality of the framework, the results already point toward a more risk-consistent and adaptable design philosophy for underground construction in weak rock.

## Supplementary Information

Below is the link to the electronic supplementary material.

Supplementary Material 1

## Data Availability

The data generated during current study are available from corresponding author, Hamid Reza Nejati, upon reasonable request (Email: h.nejati@modares.ac.ir).
